# *Candida albicans* aspects of binary titanium alloys for biomedical applications

**DOI:** 10.1093/rb/rbz052

**Published:** 2020-01-25

**Authors:** Shuyang Chen, James K H Tsoi, Peter C S Tsang, Yeong-Joon Park, Ho-Jun Song, Jukka P Matinlinna

**Affiliations:** 1 Dental Materials Science, Division of Applied Oral Sciences & Community Dental Care, Faculty of Dentistry, The University of Hong Kong, Prince Philip Dental Hospital, 34 Hospital Road, Hong Kong SAR, People’s Republic of China; 2 Department of Prosthodontics, Tianjin Stomatological Hospital, No. 75, Dagu Road, Heping District, Tianjin 300041, People's Republic of China; 3 Division of Restorative Dental Sciences, Faculty of Dentistry, The University of Hong Kong, Prince Philip Dental Hospital, 34 Hospital Road, Hong Kong SAR, People’s Republic of China; 4 Department of Dental Materials and MRC for Hard-tissue Biointerface, School of Dentistry, Chonnam National University, Gwangju 61186, Republic of Korea

**Keywords:** titanium, binary titanium alloy, *C. albicans*, fungal infection, medical devices

## Abstract

Titanium and its alloys are widely used in biomedical devices, e.g. implants, due to its biocompatibility and osseointegration ability. In fact, fungal (*Candida* spp.) infection has been identified as one of the key reasons causing the failure of the device that is inevitable and impactful to the society. Thus, this study evaluated the surface morphology, surface chemical composition and *Candida albicans* adhesion on specimens of 16 binary Ti-alloys (∼5 wt% of any one of the alloy elements: Ag, Al, Au, Co, Cr, Cu, Fe, In, Mn, Mo, Nb, Pd, Pt, Sn, V and Zr) compared with cp-Ti, targeting to seek for the binary Ti-alloys which has the lowest *C. albicans* infection. *Candida albicans* cultures were grown on the specimens for 48 h, and colony forming units (CFUs) and real-time polymerase chain reaction (RT-PCR) were used to evaluate the biofilm formation ability. Scanning electron microscopy and confocal laser scanning microscopy confirmed the formation of *C. albicans* biofilm on all specimens’ surfaces, such that CFU results showed Ti-Mo, Ti-Zr, Ti-Al and Ti-V have less *C. albicans* formed on the surfaces than cp-Ti. RT-PCR showed Ti-Zr and Ti-Cu have significantly higher *C. albicans* DNA concentrations than Ti-Al and Ti-V (*P* < 0.05), whereas Ti-Cu has even showed a statistically higher concentration than Ti-Au, Ti-Co, Ti-In and Ti-Pt (*P* < 0.05). This study confirmed that Ti-Mo, Ti-Zr, Ti-Al and Ti-V have lower the occurrence of *C. albicans* which might be clinically advantageous for medical devices, but Ti-Cu should be used in caution.

## Introduction

Given the excellent mechanical properties and biocompatibility, titanium (Ti) and its alloys has become one of the most widely used materials for biomedical devices [1–4] as non-implantable (e.g. catheter [5], tweezer and scissors [6], dental partial dentures [7], dental bridges [8], bars and attachments [9] and orthodontic wires and appliances [10, 11]) and implantable devices (e.g. artificial joints [12], bone screw [13], fixation plate [14], pacemakers [15], scaffolds [16–18] and dental implants [4, 19–21]). Indeed, Ti can form a stable oxide layer, i.e. Ti oxides (TiO_x_), as 3- to 10-nm thick film spontaneously with atmospheric oxygen and moisture. The stable ultrathin film could separate the bulk Ti material from its surrounding, and thus Ti has a high ability to resist the corrosion. Besides, the surface TiO_x_ have shown to promote the exchange of calcium, cyclic nucleotides and inositol phosphates through the gap junction channels of osteoblasts [22–24]. So, this forms the basis of why Ti is able to bond with osteoblasts and is a good choice for intraosseous applications.

Even though Ti has shown numerous advantages, it also proceeded with some other drawbacks, e.g. low wear resistance, low deformability and high reactivity with surrounding impurities, namely oxygen and nitrogen, at elevated temperatures [25, 26]. In particular to commercially pure Ti (cp-Ti), since it has a high melting point, it could not be processed easily by casting or additional manufacturing (a.k.a. 3D printing). The successful clinical case is rare [14]. Additionally, the bioactivity of TiO_x_ on the Ti surface allows not only the useful cells such as osteoblasts and proteins, but also bacteria [27, 28] and yeast [29–33] to attach. For instance, *Candida albicans* is the major infectious yeast specie in medical devices such as catheters, joints, implant and dentures [34–36]. Study has shown in USA, *C. albicans* accounted for 15% of hospital-acquired sepsis cases, which are the fourth most frequent cause of blood stream infections largely due to medical devices infection [37]. The mortality rate due to this yeast-related infection in hospital was about 40% [38]. In fact, *C. albicans* is able to form biofilm alone on Ti surface, and even provide a hypoxic microenvironment which is a ‘haven’ to anaerobic bacteria, i.e. yeast and bacteria can positively to grow together [38]. Attempts such as drug coating [31] and silane coating [32] were tried to reduce the *C. albicans* on Ti with limited success, because the coatings can be dissolved under water. Furthermore, the drug coating would induce drug-resistance that might not be useful for long-term [20]. Therefore, yeast infection by medical device is a significant problem and is a hazard for human health and healthcare system.

To achieve (or improve) the mechanical properties with balancing a good biocompatibility and keeping low virulence of yeast, alloying Ti with a variety of elements might be a viable option. Ti-alloys are sensitive to their phases/crystal structures, and stabilization of certain phases could be done by adding some alloying elements. The addition of the alloying elements can adjust the phase compositions (i.e. α, β and α−β) that change the bulk Ti-alloy properties. Thus, the mechanical properties of Ti might be enhanced and adjusted through alloying [39], such as increase the corrosion resistance, lower the modulus of elasticity and improve the machinability. Liu *et al.* has recently reviewed the mechanical properties, microstructure, chemical composition and processing of various binary Ti-alloys, and identified some metals can serve for the alloying elements, such as Al, Ag, V, Mn, Cr, Zr, Nb, Mo, Cu, In, Sn, Au, Pd and Pt [39]. Binary Ti-alloys have become a hotspot because two of the binary alloys have been commercialized: Roxolid^®^ (Straumann, Basel, Switzerland), which is a dental implant based on Ti-Zr, and Ti-15Mo (Synthes, USA), which is used as orthopedic implant.

Furthermore, Park *et al.* [40] and Song *et al.* [41] evaluated the biocompatibility of various alloying elements in binary Ti-alloys. The studies revealed that the cytotoxicity of pure metals ranked in the order of: Al > Ag > V > Mn > Cr > Zr > Nb > Mo > cp-Ti [40] and Cu > In > Ag > Cr > Sn > Au > Pd > Pt > cp-Ti [41]. All the binary Ti-alloys from 5 to 20 wt% of alloy elements except Ti-10V have statistically similar biocompatibility with cp-Ti. Therefore, alloying with Ti might be beneficial to make a better material with superior mechanical and biological performance. The objective of this study was to test and evaluate the *C. albicans* aspects on binary Ti-alloys. The hypothesis was the types of Ti-alloys would have no significant effect on the *C. albicans* adhesion.

## Materials and methods

### Ti and alloys

Sixteen types of Ti-based alloys (with ∼5 wt% of any one of the alloy elements: Ag, Al, Au, Co, Cr, Cu, Fe, In, Mn, Mo, Nb, Pd, Pt, Sn, V and Zr) and cp-Ti specimens were kindly supplied by Chonnam National University, South Korea, using a previously reported protocol [40, 41]. In brief, ∼5 wt% of pure alloy metals were homogenized with Ti metal for 4 h at temperatures 150°C using vacuum arc melting technique under a high purity argon atmosphere on a water-cooled hearth. The mass was heated below to the respective alloy’s solidus temperature and then cooled to 600°C at a rate of 10°C/min before air-cooling to room temperature. The disc-shaped specimen were then cut into diameters in ∼10 mm (Table 1) and measured by caliper. The disk surfaces were polished successively through 4000-grit SiC abrasive papers, and ultrasonically cleaned in acetone, ethanol and distilled water, before the following tests.

**Table 1:** The wt% of alloying element and diameter for each Ti-alloy

Sample	Alloying element (wt%)	Mean diameter (mm)
Ti-5Ag	5.04	9.97
Ti-5Al	4.75	9.97
Ti-5Au	5.26	10.00
Ti-5Co	5.67	9.98
Ti-5Cr	4.59	9.99
Ti-5Cu	4.72	9.64
Ti-5Fe	5.47	9.93
Ti-5In	4.68	9.95
Ti-5Mn	5.02	9.95
Ti-5Mo	5.22	9.60
Ti-5Nb	5.78	10.00
Ti-5Pd	5.77	9.95
Ti-5Pt	4.61	9.92
Ti-5Sn	5.60	9.90
Ti-5V	4.37	9.96
Ti-5Zr	4.89	9.96
cp-Ti	N/A	9.79

### Surface analyses

#### Scanning electron microscopy

To observe the morphology, one of the specimens from each group was prepared for scanning electron microscopy (SEM; Hitachi SU-1510 [VP-SEM, Tokyo, Japan]). They were fixed on aluminum stubs, and then observed with the acceleration voltage 15 kV in high-vacuum mode and the height of the electrode was ∼15 mm. 500× and 2000× magnifications were used to overview and observe the initial condition of the specimen surfaces, and biofilm formation after the culture (Culturing of *C. albicans* and biofilm development section), respectively. Three different points from each specimen were chosen to record.

To observe the biofilm, specimens were fixed by immersing them for 1.5 h in 2.5% glutaraldehyde (BDH Lab. Supplies, UK). Subsequently, they were dehydrated by putting into a series of ethanol (70%, followed by 85%, 95% for once and absolute for twice) for 15 min. Finally, the dry specimens were sputtered-coated with Pd-Pt-coating.

#### Energy-dispersive X-ray spectroscopy

The chemical compositions of the 16 types of Ti-alloys and cp-Ti were analyzed with energy-dispersive X-ray spectroscopy (EDX) module (IXRF systems, Inc., Austin, TX, USA) that was mounted on the SEM. Three different spectra on the surface of the sample were analyzed with EDX. The silicon EDX detector was used, together with the accelerating voltage 15.00 kV in high-vacuum mode. The magnification 500× was used and the area of observed spectra was ∼500 × 500 μm^2^. No element was excluded from the analysis and the number of iterations was six.

### Microbiology

#### Sample allocation, pretreatment and post-treatment

For the microbiological tests, the cp-Ti was the control group, while the 16 types of Ti-alloys were the test groups. Before the culture, the Ti-alloys and cp-Ti were polished by 4000-grits SiC abrasive paper and pre-cleaned in an ultrasonic bath (Decon FS200; Decon Ultrasonics Ltd, Hove, UK) strictly following the cleaning protocol [(i) 95% ethanol for 5 min; (ii) Deionised (DI) water for 3 min; and (iii) acetone for 5 min] before each culture. This has been pretested in-house that could completely remove the remnant biofilm and dirts (if existed). After the ultrasonic cleaning, the specimens were rinsed by the DI water and then steam autoclaved (Autoclave ASB300BT, ASTELL, UK) at 121°C for 15 min [32, 42]. After that, these sterilized specimens were put into sterile 24-well plates (Iwaki, Tokyo, Japan).

#### *Culturing of* C. albicans *and biofilm development*

The *C.* *albicans* (ATCC 90028) was cultured on Sabouraud dextrose agar (Gibco, Paisley, UK) for 18 h at 37°C. Then, the cells were washed in phosphate-buffered saline (PBS) at pH 7.2 for twice and the pellet was harvested by centrifugation at 4000 rpm (GS-15R centrifuge; Beckman Instruments Inc., CA, USA) for 10 min. The cell suspension was prepared with 100 mM glucose and yeast nitrogen base (Difco Laboratory Inc., USA) at McFarland scale 4.

After the standardization of the culture, 1.5-ml cell suspension was added in wells to ensure a full coverage of the cell suspension on the materials surface. Then, the plate was kept at 37°C, 80 rpm in a shaking incubator (Stuart SI500, Bibby Scientific Ltd., Staffordshire, UK) for 90 min. After that, the media were refreshed and further kept for 48 h with the media were changed after 24 h. After 48 h biofilm culturing, the specimens were washed in PBS and transferred to a new well plates that has 1.0 ml PBS for each well. Then, the attached biofilm for each specimen was rinsed out by repeated pipetting.

#### Colony forming units

Colony forming units (CFUs) is a possible method to quantify the amount of living cells in a solution. In this study, for each group, the collected cell suspension from each well was diluted in the factors of 10^−^^2^, 10^−^^3^ and 10^−^^4^. Then, 50 μl (i.e. 0.05 ml) for each diluted cell suspension was plated on Sabouraud agar using a spiral plater (Autoplate 4000; Spiral Biotech Inc., Bethesda, MD, USA). The plates were kept at 37°C incubator for 48 h. The rationale for various dilution factors is to obtain plates with colony counts between 30 and 300. If the counts were smaller or larger than these range, than the counts from another dilution factors would be used. The CFU/ml for each group was calculated by:

$$\text{CFU}/\text{ml}=\frac{\left(\text{no}.\;\text{of}\;\text{colonies}\times \text{dilution}\;\text{factors}\right)}{\text{volume}\;\text{plated}\;\text{in}\;ml\;}$$

For this case, the volume plated is 0.05 ml. Furthermore, since each specimen might have various diameters; therefore, the presented results are as per unit area (mm^2^). Three attempts were tried for each group.

#### Real-time polymerase chain reaction

To work out the real-time polymerase chain reaction (RT-PCR), the first procedure was to extract the DNA of *C. albicans* according to DNA extraction kit procedure. In brief, firstly, harvested cells from Culturing of *C. albicans* and biofilm development section for each sample were obtained by centrifugation (Sorvall Legend Micro 21 centrifuge; Thermo Electron LED GmbH., Osterode, Germany) at full speed (14 500 rpm) for 1 min. Then, the supernatant was removed and the solid residues were resuspended with 293 μl 50 mM ethylenediaminetetraacetic acid in eppendorf tube. A 15 μl lyticase (20 mg/ml) was added into each tube and they were incubated in a water bath (Jalabo TW12; Jalabo Labortechnik GmbH., Seelbach, Germany) at 37°C for 1 h. After that, the tubes were centrifuged at 13 000 rpm for 5 min. Again, the supernatant was discarded, but this time the spheroplasts were resuspended with the 180 μl tissue lysis buffer (Buffer ATL; QIAGEN GmbH, Hilden, Germany). A 20 μl proteinase K was added into each tube and vortexed, and the tubes were incubated in a water bath at 56°C for 10 min. Additionally, 200 μl lysis buffer (Buffer AL; QIAGEN GmbH., Hilden, Germany) was added to the samples and vortexed for 15 s. The tubes were then incubated in a water bath at 70°C for 10 min. A 200 μl absolute ethanol was then added and vortexed. The samples were applied carefully to the spin columns (QIAGEN GmbH., Hilden, Germany) without wetting the rim. The spin columns were centrifuged at 8000 rpm for 1 min and then replaced with a clean 2 ml collection tube. Five hundred microliters of wash buffer 1 (Buffer AW1; QIAGEN GmbH., Hilden, Germany) were added to the columns without wetting the rim. Then, the columns were centrifuged at 8000 rpm for 1 min and replaced with a clean 2 ml collection tube. Five hundred microliters wash buffer 2 (Buffer AW2; QIAGEN GmbH., Hilden, Germany) were added to the columns. These columns were centrifuged at 14 000 rpm for 3 min, again replaced with a clean 2 ml collection tube and centrifuged again at full speed for 1 min. Finally, the columns were transferred to a new 1.5 ml eppendorf tubes, with addition of 100 μl elution buffer (Buffer AE; QIAGEN GmbH., Hilden, Germany), then incubated at room temperature for 5 min with final centrifugation at 8000 rpm for 1 min.

After these procedures, the DNA of *C. albicans* was extracted and collected, and then stored in the eppendorf tubes (‘cell solution’) at −21°C until the RT-PCR was carried out. On the other hand, the ‘master mix’ solution was prepared containing 5 μl of a nucleic acid stain (QuanitFast SYBR Green; QIAGEN Gmbh., Hilden, Germany), 2 μl DI water, 1 μl forward primer (5ʹ GGG TTT GCT TGA AAG ACG GTA 3ʹ) and 1 μl reverse primer (5ʹ TTG AAG ATA TAC GTG GTG GAC GTT 3ʹ).

To quantify the amount of *C. albicans* by RT-PCR, firstly standard curves by using known cell-concentrations solutions containing 10^8^, 10^7^, 10^6^, 10^5^, 10^4^ and 10^3^ cells of *C. albicans* have been generated by software (StepOne Software V2.2). Then, in separate wells of a 0.1 ml well plate (MicroAmp Fast optical 96-Wellplate; Applied Biosystems Pty Ltd., Scoresby, Australia), 1 μl of ‘cell solution’ was mixed with 9 μl ‘master mix’. The plate was covered with a cohesive cover (MicroAmp optical adhesive Film; Applied Biosystems Pty Ltd., Scoresby, Australia) and analyzed by a PCR machine (StepOnePlus; Applied Biosystems Pty Ltd., Scoresby, Australia). Each group was analyzed for six times.

#### Confocal laser scanning microscopy

The 48 h biofilm obtained from each specimen in Culturing of *C. albicans* and biofilm development section was washed with PBS, and proceed with confocal laser scanning microscopy (CLSM). Then, they were stained by the LIVE/DEAD bacterial viability kit (Molecular Probes L7012) for half an hour and then observed under CSLM (IX81S1F-3, Olympus, Tokyo, Japan) using different magnifications. The pictures were analyzed by the equipment’s software (FV10-ASW 3.1 Viewer).

### Statistical analysis

CFU and DNA concentrations (by RT-PCR) were statistically analyzed by using Kruskal–Wallis *H* test and one-way ANOVA, respectively (IBM SPSS Statistics for Windows, Version 22.0. Armonk, NY: IBM Corp.).

## Results

### Materials characterization

**Figure 1:**
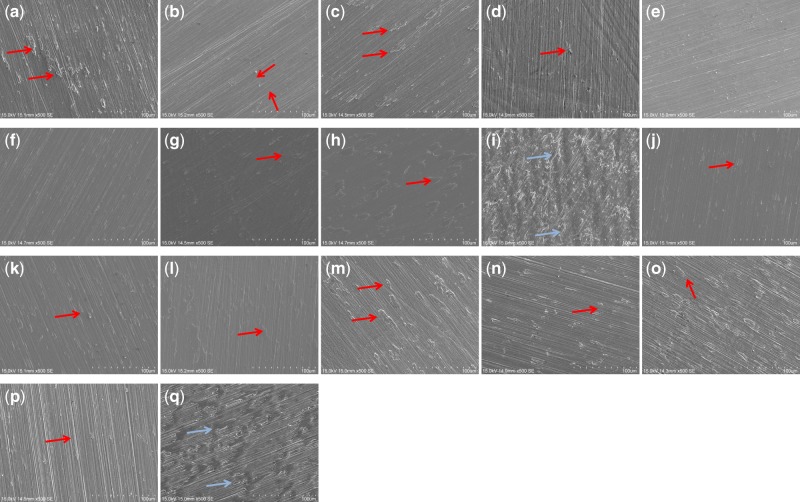
SEM micrographs of the polished Ti-alloys and cp-Ti at 500× magnification. **(a)** Ti-5Ag, **(b)** Ti-5Al, **(c)** Ti-5Au, **(d)** Ti-5Co, **(e)** Ti-5Cr, **(f)** Ti-5Cu, **(g)** Ti-5Fe, **(h)** Ti-5In, **(i)** Ti-5Mn, **(j)** Ti-5Mo, **(k)** Ti-5Nb, **(l)** Ti-5Pd, **(m)** Ti-5Pt, **(n)** Ti-5Sn, **(o)** Ti-5V, **(p)** Ti-5Zr and **(q)** cp-Ti. Red arrows indicate the flaws and blue arrows indicate the microcracks

The morphological structure of the 16 types of binary Ti-alloys and cp-Ti was observed by SEM (Fig. 1) with 500× magnification. The polished Ti-alloys and cp-Ti surfaces are homogenous, slightly scratched with regularly distributed flaws in various size and shape. In particular, Ti-5Mn and cp-Ti have shown small microcracks (i.e. crevices).

Table 2 shows the EDX elemental analysis for the specimens after polished with SiC abrasive paper. In particular, Ti, C, O and the alloying elements have been evaluated. All the tested binary Ti-alloys well correspond to the information as per supplied (Table 1). Minute amount of Si and C was detected due to the use of SiC abrasive paper to polish the specimen in Ti-alloys and cp-Ti.

**Table 2:** EDX analysis (wt%) of the specimen after polished with SiC abrasive paper, X represents the alloying elements

Sample	Ti	C	O	X	Si
Ti-5Ag	91.327	0.985	0.000	6.521	1.167
Ti-5Al	86.518	0.882	5.453	6.116	1.032
Ti-5Au	87.173	0.892	6.469	4.303	1.163
Ti-5Co	86.216	0.576	3.772	7.756	1.681
Ti-5Cr	89.041	1.306	1.599	6.331	1.723
Ti-5Cu	88.051	0.773	3.491	5.587	2.098
Ti-5Fe	86.408	0.581	3.921	7.783	1.306
Ti-5In	87.091	0.986	4.894	6.066	0.962
Ti-5Mn	84.356	0.151	6.803	7.015	1.674
Ti-5Mo	89.456	0.277	3.050	5.950	1.267
Ti-5Nb	87.446	0.000	4.943	6.427	1.184
Ti-5Pd	89.776	2.620	0.000	6.595	1.009
Ti-5Pt	89.219	0.415	4.958	4.489	0.920
Ti-5Sn	87.226	0.338	3.761	7.333	1.342
Ti-5V	86.853	0.157	6.337	5.436	1.216
Ti-5Zr	86.747	0.308	5.854	6.466	0.900
cp-Ti	91.365	0.404	6.289	N/A	1.942

X corresponds to the alloying elements.

### Microbiology

**Figure 2:**
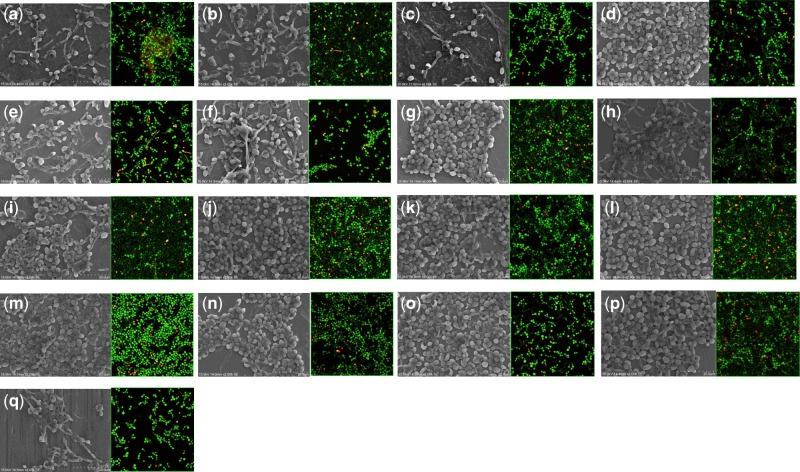
SEM micrographs (2000×) magnification and CLSM of the stained *candida* on the specimen **(a)** Ti-5Ag, **(b)** Ti-5Al, **(c)** Ti-5Au, **(d)** Ti-5Co, **(e)** Ti-5Cr, **(f)** Ti-5Cu, **(g)** Ti-5Fe, **(h)** Ti-5In, **(i)** Ti-5Mn, **(j)** Ti-5Mo, **(k)** Ti-5Nb, **(l)** Ti-5Pd, **(m)** Ti-5Pt, **(n)** Ti-5Sn, **(o)** Ti-5V, **(p)** Ti-5Zr and **(q)** cp-Ti. Alive cell was stained in green, and dead cell was stained in red

**Figure 3:**
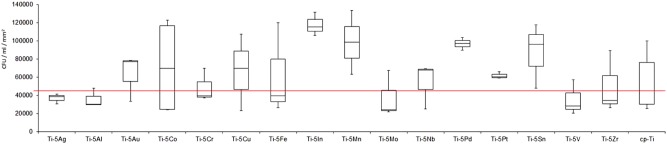
Box-plot (min–max, box: Q1–Q3) of the CFU/ml per unit area in this study. Red line indicates the cp-Ti value for easier comparison

After the 48 h biofilm formation, SEM and CLSM (Fig. 2) were used to observe the attachment of the biofilm. *Candida* *albicans* could grow on all the surfaces. Figure 3 illustrated the median CFU/ml per unit area and the mean rank of the materials. When compared with the cp-Ti, with the alloy elements Mo, V, Al, Zr, Ag, Cr and Fe would give a lower prevalence of *C. albicans* adhesion in CFU/ml per unit area (mm^2^) on Ti-alloys. In general, the ascending order of *C. albicans* prevalence is:

Ti-Mo < Ti-V < Ti-Al < Ti-Zr < Ti-Ag < Ti-Cr < Ti-Fe < cp-Ti < Ti-Pt < Ti-Nb < Ti-Co < Ti-Cu <Ti-Au < Ti-Sn < Ti-Pd < Ti-Mn < Ti-In.

However, Kruskal–Wallis *H* test showed that there was no statistically significant difference in CFU/ml/mm^2^ (χ^2^ = 21.96, *P* = 0.144) between groups.

**Figure 4:**
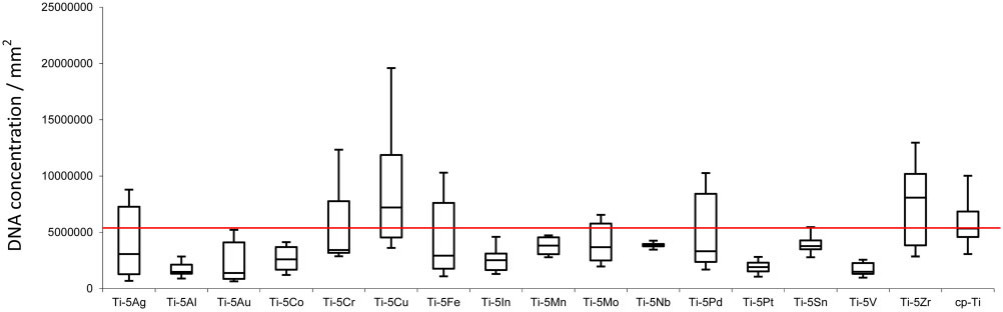
Box-plot (min–max, box: Q1–Q3) of the *C. albicans* DNA concentration per unit area in this study. Red line indicates the cp-Ti value for easier comparison

RT-PCR results are shown in Fig. 4. PCR results revealed that only Ti-Cu and Ti-Zr groups have a higher *C. albicans* DNA concentration (per unit area) than cp-Ti. One-way ANOVA showed the DNA concentrations are highly statistically significant (*P* ∼ 0.0004 < 0.01) between the groups, whereas Tukey HSD *post hoc* test Ti-Al and Ti-V have statistically lower DNA concentrations than Ti-Cu (*P* < 0.05) and Ti-Zr (*P* < 0.05). Ti-Cu also showed a high DNA concentration than Ti-Au, Ti-Co, Ti-In and Ti-Pt (*P* < 0.05).

## Discussion

To the best of authors’ knowledge, this is the first study to compare the *C. albicans* biofilm formation ability on binary Ti-alloys. This study successfully cultured the biofilm on all the binary Ti-alloys and cp-Ti. Thus, it might also implies, if such Ti and alloys are used as medical devices, biofilm are possible to form on the surface. This study revealed that some alloying elements Mo, V, Al, Zr, Ag, Cr and Fe might give a lower CFU than cp-Ti, but not statistically significant. RT-PCR revealed that Ti-Cu seems to give the highest total *C. albicans* DNA concentration than others, and Ti-Zr surprisingly ranked the second, both are statistically higher than the lowest Ti-Al and Ti-V groups.

Fundamentally, RT-PCR and CFU are measuring different parameters and performance of the biological sample, which contains living (shown as green in Fig. 2 CLSM) or dead cells (shown as red in Fig. 2 CLSM). For CFU, the method is related to culture the *C. albicans* on agar plate, thus the measurement is related to those bacterium still alive. For RT-PCR, the method for detection is related to bind the DNA fragments of the cells with the primers, regardless the cells are living or dead. As a consequence, RT-PCR detects the total cell number.

Furthermore, PCR utilized for the fluorescent emission technique which is very sensitive for the pH of the solution. As mentioned in Han *et al.* [20] and Liu *et al*. [4], in particular for Ti surface, at the atmospheric environment, small portion of Ti and Ti-OH at the surface would chemically react (by chemisorption) with the water moisture which might lead weakly bounded physisorbed water on the surface. In the condition of multivalent Ti (e.g. Ti^4+^) metal, together with the physisorbed water that proceeds with the equilibrium dissociation reaction and becomes hydroxide (OH^−^) and hydronium (H^+^) ions, Ti is ready to form Ti-OH:

(1) $$H_{2}O\;↔{\;H}^{+}+{\;\text{OH}}^{-}$$

(2) $${\text{Ti}}^{4+}+{\;4\text{OH}}^{-}↔\;4\text{Ti}-\text{OH}$$

Then, the Ti-OH is likely to undergo further hydrolysis [43]:

(3) $$\text{Ti}-\text{OH}+{\;H}_{2}O\;↔\;{\left[\text{Ti}-O\right]}^{-}+{\;H}_{3}O^{+}$$

(4) $$\text{Ti}-\text{OH}+{\;H}_{2}O\;↔\;{\left[\text{Ti}-{\text{OH}}_{2}\right]}^{+}+{\;\text{OH}}^{-}$$

In theory, equilibrium reaction (3) would lead to the formation of basic type of TiO_x_ ([Ti-O]^−^) and the acidic type [Ti-OH_2_]^+^. These TiO_x_ species are in nano thickness. Studies [44–46] had shown that the isoelectric point (IEP) for these TiO_x_ at surface ranged from 5.0 to 6.7. The equilibrium reactions (3) and (4) suggested that, for environment pH is more acidic, i.e. lower pH, than IEP, the predominant oxide specie would be [Ti-OH_2_]^+^, and vice versa. Indeed, from Table 2, we knew that binary Ti-alloys (except Ti-Pd) and cp-Ti contains certain oxides. Thus, alloying Ti with various elements (and even to different alloying percentage [47]) would constitute different oxides and TiO_x_ content, i.e. various IEP. This said, the pH of the bacterium medium in Culturing of *C. albicans* and biofilm development section might be changed, such that the fluorescent emission of commercial PCR reagents might not correspond to the set standard curve (in *Real-time polymerase chain reaction* section). Therefore, to improve the RT-PCR experiment, the pH from each sample bacterium medium should be determined and standard curves in various pH should be drawn.

For Ti-Al and Ti-V, the biocompatibility was determined to be statistically comparable with cp-Ti [40]; however, the leaching of Al and V is inevitable [48]. Therefore, the release of these metal particles or ions could poison the *C. albicans*, because they possibly inhibit the formation of cell by interrupting the cell DNA synthesis. Hence, we could not find a high CFU values in Ti-Al and Ti-V, and the total *C. albicans* DNA concentration is less than others. In noble metals alloying elements, i.e. Ti-Ag, Ti-Pd, Ti-Pt and Ti-Au, they also have low DNA concentrations of *C. albicans* but a high CFU values, except for Ti-Ag. Pd, Pt and Au are bioinert that would not kill *C. albicans*. However, Ag has been demonstrated anti-bacterial effect due to its ‘zombie’ ability [49], whilst this anti-bacterial effect will need to happen in its cationic state. Thus, such a phenomenon might mean Ag from the alloy surface is releasing in Ag^+^ form, and the material mechanical properties might be affected. Further clarification is necessary.

Ti-Mo has been used as one of the commercialized orthopedic implant (Ti-15Mo, Synthes, USA), which performed the lowest CFU in this study. However, a careful selection about the percentage of Mo is necessary, since the existence of ω phase [50] at low concentration of Mo (<15%) might have low temperature ω → α transformation and thus affect the materials strength.

Ti-Zr, on the other hand, has been marketed as Roxolid^®^ dental implant (Straumann, Basel, Switzerland) in ∼15% Zr. This study has shown Ti-Zr has the highest count on *C. albicans* concentration, but a relatively a low CFU count than cp-Ti and among others except for Al, V and Mo. Indeed this is an attractive Ti-alloy that posses with excellent biocompatibility and strength for biomedical applications. However, the processing is a challenge [51], i.e. for low concentration of Zr, the strength and elastic recovery (i.e. springback) properties for dental applications might not be sufficient due to the necessity of a harsh processing environment to reduce as much oxygen as possible. Nonetheless, the chemical similarity between Ti and Zr and acid etching ability would outcome all these environmental factors, such that Ti-Zr should pertain its foreseeable and fruitful future.

Ti-Cu has illustrated both high CFU and *C. albicans* DNA concentrations. Despite it might exhibit a good mechanical grindability and wear resistance [52, 53] that is good for CAD/CAM denture application [54], as well as good osteogenic and biocompatibility [55], the risk of *Candida* infectious contamination is high. Thus, cautious should be paid when using Ti-Cu as an implantable biomaterial in medical device.

## Conclusion

Different types of binary Ti-alloys would not significantly affect the *C. albicans* adhesion is rejected. Binary Ti-alloys with 5 wt% of Mo, Al, V, Zr could reduce the occurrence of *C. albicans* which might be clinically advantageous for medical devices.
